# Vicious circles of gender bias, lower positions, and lower performance: Gender differences in scholarly productivity and impact

**DOI:** 10.1371/journal.pone.0183301

**Published:** 2017-08-25

**Authors:** Peter van den Besselaar, Ulf Sandström

**Affiliations:** 1 Vrije Universiteit Amsterdam, Network Institute & Institute for Societal Resilience, Amsterdam, The Netherlands; 2 KTH Royal Institute of Technology, INDEK, Stockholm, Sweden; Universidad de las Palmas de Gran Canaria, SPAIN

## Abstract

It is often argued that female researchers publish on average less than male researchers do, but male and female authored papers have an equal impact. In this paper we try to better understand this phenomenon by (i) comparing the share of male and female researchers within different productivity classes, and (ii) by comparing productivity whereas controlling for a series of relevant covariates. The study is based on a disambiguated Swedish author dataset, consisting of 47,000 researchers and their WoS-publications during the period of 2008-2011 with citations until 2015. As the analysis shows, in order to have impact quantity does make a difference for male and female researchers alike—but women are vastly underrepresented in the group of most productive researchers. We discuss and test several possible explanations of this finding, using a data on personal characteristics from several Swedish universities. Gender differences in age, authorship position, and academic rank do explain quite a part of the productivity differences.

## Introduction

In the scientific enterprise, the few extremely prolific researchers play an important role [1]: the “superstars” who publishes less in isolated areas, in dying areas, or in areas without inherent dynamics, and more in upcoming, central and dynamic areas. Highly productive and cited researchers tend to look for new opportunities, and they play a crucial role in science [2]. It is well known that a strong correlation exists between productivity (number of papers) and overall impact (number of citations), but does this also hold for the important papers: high productive researchers are also producing the bulk of the important (highly cited) publications. The more papers, the more high-impact papers, and the larger the share of high impact papers [3; 4]. Not much research is done on the relation between productivity and top papers, but these scarce studies point in the same direction as our findings [5; 6].

Given this overall relation between productivity and the production of top cited papers, does this equally hold for male and female researchers? Quite some literature exists on gendered publication differences, and it is in the mean-time a well-known and widely accepted observation that male researchers are more productive than female researchers. The issue of performance differences between male and female researchers remains important in relation to the ongoing debate about gender bias in science. Are these performance differences a consequence or an antecedent of the unequal position in women in the science system? For a more extensive overview of the literature on this issue we refer to an earlier paper of one of us [7]. In Table 1, we briefly summarize a part of the literature to show the different methodological choices and variables included (c.f. [8] p211).

**Table 1:** Overview of approaches with example studies.

Authors	Level	Country coverage	More disciplines	Specific cohorts	Sample size	Data /Method	Period covered
Xie & Schauman 1998 [9]	Person	US	Yes	Yes	Large	Survey*/**	1969–1993
Nakhaie 2002 [10]	Person	Canada	Yes	Yes	Large	Survey**	n.a. -1987
Symonds 2006 [12]	Person	Australia	No	Yes	Small	WoS	1990–2005
		UK				Council data	
van Arensbergen 2012 [7]	Person	Netherlands	No	Yes	Small	WoS	2003–2010
						Council data	
						WWW	
West et al. 2013 [13]	Paper	International	Yes	No	Large	JSTOR	1990–2011
Sugimoto et al. 2013 [8]	Paper	International	Yes	No	Large	WoS	2008–2012
Cameron et al. 2016 [11]	Person	International	No	Yes	Large	Scopus	1994–2008
Beaudry & Lariviere 2016 [14]	Person	Canada	Yes	No	Large	WoS	2000–2012

* included items about funding;

** Self-reported productivity;

n.a. = unknown

The older studies with large samples and covering more disciplines [9; 10] were based on surveys, with the disadvantage that performance is based on self-reporting. Other studies went to WoS data for measuring performance and thereby used the *paper* as unit of analysis, and assigned gender based on first name. This approach, however, does not really allow for productivity analysis on the *individual* level. After 2012, new disambiguation techniques [11] for bibliometric data and for author identification enabled the use of individual researchers as unit of analysis also in large-scale studies. Several but not all newer studies have been able to go beyond the country-specific and single-discipline approaches. However, some of these studies are based on cross-sectional data, and therefore mixing individuals of different age cohorts and positions—which may seriously affect the findings.

Furthermore, most studies rely on relatively small samples [12], restricting the generalizability of the findings. A notable exception is the classic study by Xie and Schauman [9] which is based on four large surveys with about 20,000 respondents over a time period between 1969 and 1993. One of the most interesting findings of their study is the change over time in the relation between gender and research productivity. In the studied period the difference in productivity went down: Whereas in 1969 female researchers’ productivity was 65% of male researchers’ productivity, in 1993 this had increased to 75%. The question is whether we can interpret this as a trend, following the increasing participation of women in science.

**Fig 1:**
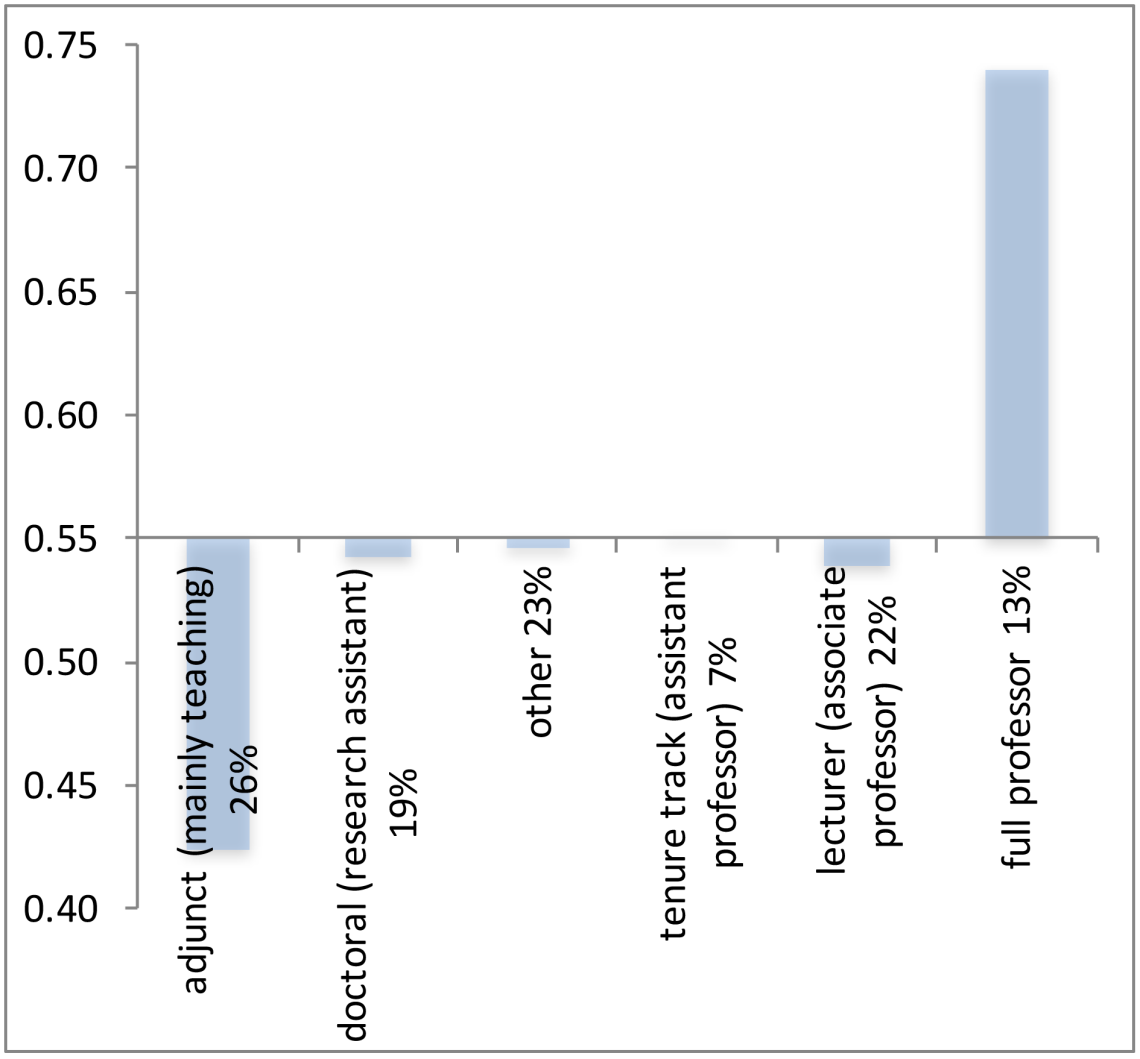
Swedish researchers by gender by age. Vertical axis: share male researchers; horizontal axis: age groups. Overall share of men: 58% (in 2008).Source: [20].

**Fig 2:**
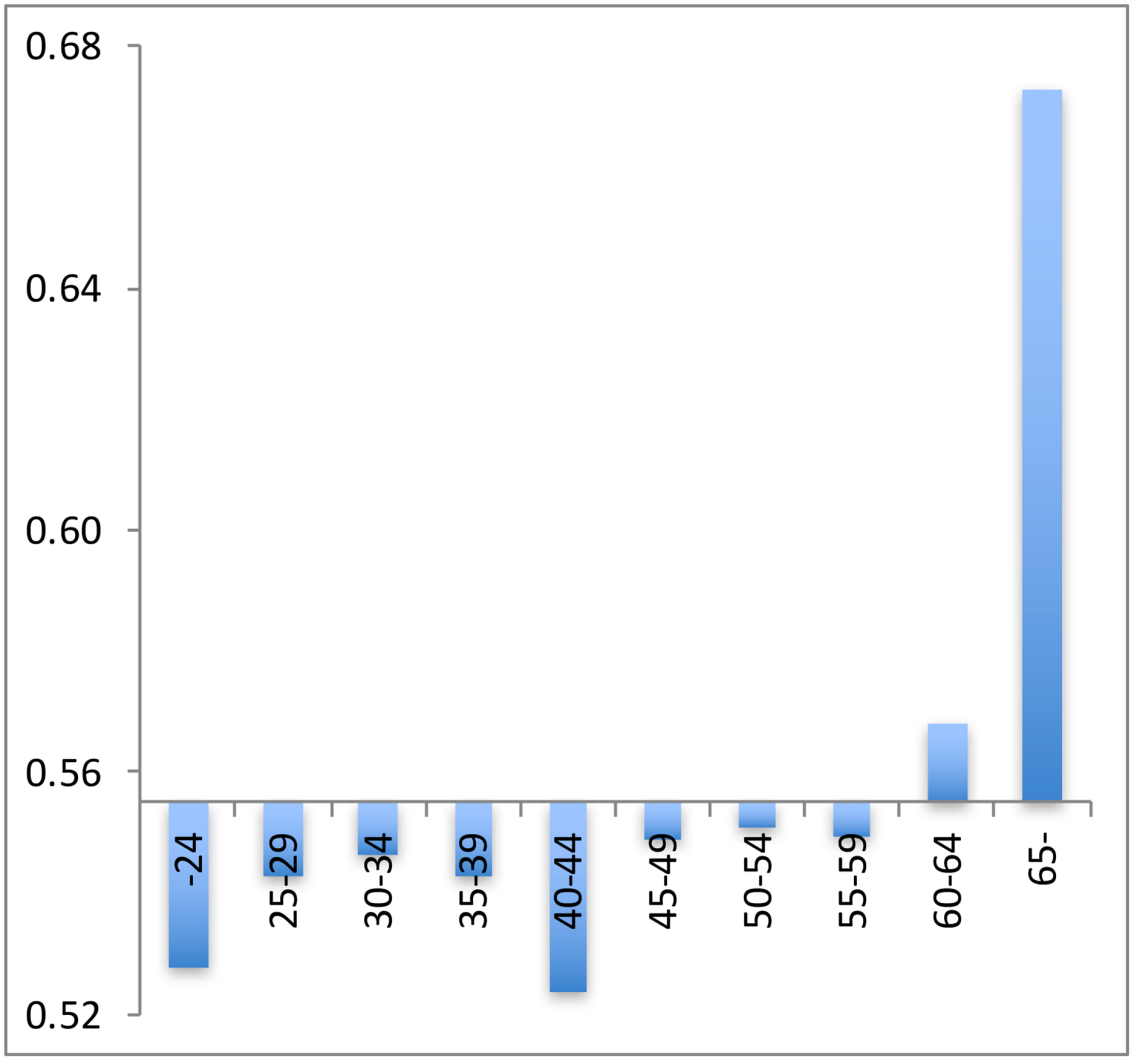
Swedish researchers by gender and job level. Vertical axis: share male researchers; horizontal axis: position ordered by increasing job level. The percentage after the job name: the share of that position within total Swedish academic workforce. Overall share of men: 55.4% (in 2015). Source: [21].

Several possible explanations of the gender differences in productivity have been suggested. (i) Female researchers are on average substantially younger than male researchers (see Fig 1), and the high productive researchers are to be found in the more senior (higher age) groups [5; 6]. If this would be the only factor, one would expect that the observed productivity differences would further decline (in line with the Xie & Schauman study [9]) and disappear over time. But also other structural and/or behavioral factors may underlie gender productivity differences, hampering female academic careers [7; 15] and leading to a waste of talent. (ii) Women are rather strongly overrepresented in the lower academic positions, and in positions with a temporary contract (Fig 2), positions which are generally characterized by a higher teaching load, less access to funding, less career perspectives, and less opportunities for research [16; 17; 18; 19]. Indeed, there is a positive relation between job level and productivity. This situation is less prone to gradual change, as it may be the effect of gender bias and of a sustained existence of the glass ceiling in academic institutions [15].

(iii) Women may have less access to research funding, whereas winning prestigious research grants is characterized by gender biased in favor of men, and above that very influential for the grant winners’ career [15; 14]. (iv) Female researchers have a lower status within teams and collaboration networks, and get less opportunities to become an independent researcher. This is reflected in different author positions on papers. Women more often get the less prestigious positions: the last author (= team leader) is more often a male researcher, whereas female researchers more often occupy ‘in between’ author positions. This may result in a slower career of female researchers compared to the career of male researchers [8; 13]. More directly, Van den Besselaar & Sandström showed that men progress faster through the various academic ranks [22]. (v) Productivity relates to the organizational environment where a researcher works [23], and if female researchers have more problems in being hired in top environments [24], this is expected to affect productivity differences between men and women.

In fact, gender differences may be the effect of a combination of these five factors. In order to investigate whether over time productivity differences are disappearing, or whether other (structural) factors avoid that from happening, one would need a reliable bibliometric dataset at the level of (disambiguated) individuals, combining data on the output (paper production, citations, author position) with data on gender, field of research, scientific age (time since PhD), and position of the researcher. Moreover, field information is necessary: we have to control for field differences in paper production levels, because of the different distribution of male and female researcher over fields. Age is important—especially when we use output data covering a specific period and performance may be influenced by the length of the career before that period. Data about the academic position is needed in order to sort out whether gender differences in productivity are partly accounted for by differences in position. Finally, one would need data on funding as output is highly dependent on grants [25]. Some studies have indicated that funding sources may explain long term differences in productivity, but these are small scale [12]. Recent large-scale studies on individual funding do not analyze gendered productivity differences [13; 8; 14]: in large scale studies it is still a problem to create unique author identities. An exception is Beaudry & Lariviere [14] but they do not report on differences in productivity.

### Research questions

In this paper we contribute to the literature explaining gender differences in research productivity by analyzing the complete set of Swedish researchers. We firstly compare male and female researchers’ performance using the seven productivity classes, for different areas of research. Then we investigate gender performance differences whereas controlling for the variables mentioned in the literature, using a sample from our dataset on Swedish universities. For this sample, we do have data on age, academic rank, field, and on last author position. By answering the following three questions, this analysis will provide a better insight in gendered performance differences in the recent period:

1. Is the relation between productivity (number of publications) and quality (number of high impact papers) different for women than for men?
2. Are male and female researchers differently distributed over the productivity classes?
3. Do age, author position, and academic position explain the observed productivity differences, whereas controlling for research field?

If the trend observed by Xie & Schauman [9] has continued, the western world should have reached gender equality in productivity (i.e. about 2005-2012). Better understanding the gender productivity differences may also contribute to the debate on the continuing underrepresentation of female researchers in getting top positions and in receiving top grants [26; 15].

## Methods and data

As we use the same data set here as in [4] and [26], the text in this section heavily draws on both mentioned papers.

### Data handling

In order to answer our questions, we use the 74,000 WoS-publications from 2008–2011 (with citations until 2014) of all researchers with a Swedish address using the following document types in databases SCI-E, SSCI and A&HCI: articles, letters, proceeding papers and reviews. For identifying authors and keeping them separate we use a combination of automatic and manual *disambiguation* methods. An algorithm for disambiguating unique individuals was developed by Sandström & Sandström [27], based on Soler [28], and further developed by Gurney et al. [29]. It proceeds fast, although it still requires some manual cleaning. The deployed method takes into account surnames and first-name initials, the words that occur in article headings, and the journals, addresses, references and journal categories used by each researcher.

The 74,000 articles consist of 195,000 author-shares (part of an article, proportional to the number of authors) in those publications with a Swedish address. In some cases we also included the articles from people who have worked both in Sweden and in another Nordic country. So disambiguating names was done at the Nordic level, as the academic labor market is to quite a large extent Scandinavian.

For each researcher, articles are ranked in terms of received citations. Then the articles are divided into CSS classes (Characteristic Scores and Scales 0,1,2,3) classes, as an alternative for percentile groups (e.g. top10% etc.). CSS have some advantages over percentile groups for identification outstanding citation classes. Details can be found in [30]. We here use level CSS1 and mainly CSS3, which cover the 37% and 3.5% most cited papers respectively. The latter can be seen as the “outstandingly cited papers” [31]. In the second part of this paper we will also deploy the top 10% most cited papers.

### Identification of unique authors

Until recently, the Web of Science was highly suitable for breaking down the data by research field, country or affiliation, but it could hardly be used for identifying individuals. Only since 2008, authors’ names are in most cases linked to an address, which facilitates the disambiguation process. Before 2008, WoS only had the address of the ‘corresponding author’.

Even if each researcher has only one name spelling variant, there remains the homonym issue: there are thousands of Swedes with exactly the same name as someone else. ID numbers used for researchers are generally based on surname and the first initial, which makes the problem even greater. E.g., according to our data, at Lund University eight persons may have as author name ‘Andersson, K.’, and in Uppsala University (Which includes SLU—the Swedish University of Agricultural Sciences) seven individuals share the name ‘Andersson, A.’ Using the algorithm method developed by Sandström & Sandström [27], persons with the same name are distinguishable if they work in different subfields. However, there are researchers who’s ‘surname—first initial combination’ can be found at six universities or more. These very common combinations have therefore been excluded from our analysis, which refers to about 1000 persons.

Moed [32] studied how a single researcher’s name can vary in Web of Science. Some variations are ‘legitimate’, generated by the researchers themselves, and others are ‘illegitimate’ because of errors such as spelling mistakes. Errors in entering the data are few and do not constitute a great problem, while ‘legitimate’ variations can cause problems when one tries to merge those to a single identity. Broadly, four types can be distinguished: direct misspellings; those arising from transcribing names from other alphabets; people changing name due to e.g., marriage or divorce; and variations in the use of initials. We have tested our method several times with randomly selected publication lists of researchers. Comparing our disambiguation method with manual adjustment procedures, we obtained agreement of more than 98%.

Since 2008, the author’s first name is added to the WoS metadata, and this is an important extension, as full first names are needed for determining gender. However, there are still journals that do not have full first names but only the initials. Partly this can be solved by assigning gender after the disambiguation procedure, as we then may have at least some papers with the first name. But, still for some authors we do not have more than the initial and those will be missing values.

To assign gender for those with first name, we have applied a combination of methods. In some cases, the last two letters of the first name reflect gender: e.g., in case of names ending with ‘-na’ or ‘-va’, which are usually women’s names (Lena, Irina, Kristina, Eva), while those ending in ‘-an’, ‘-av’, ‘-rs’ or ‘-lf’ (Håkan, Gustav, Anders, Rolf, Ingolf) are men’s names. This procedure, with simultaneous manual checks, enabled to assign gender to the majority of names in the Swedish dataset. Further information is available in name databases, such as the US Census, Wiki-names and Wikipedia. Sugimoto and colleagues [8] deployed that method to assign gender to WoS authors of more than five million papers published between 2008 and 2012: they were able to assign gender to about 65% of the author shares. It should be emphasized that their method works at the paper level, as they did not try to identify unique authors. The combination of methods in this paper has increased the coverage considerably above that percentage (see below).

To further improve assigning gender for the Swedish dataset, we also did manual matching using different resources. Firstly, we used staff databases obtained from the major Swedish universities. Secondly, we deployed SwePub (swepub.kb.se), containing publications with full authors’ names. Thirdly, we searched for Asian authors names on the Internet, especially the names of Chinese, Japanese, Iranian and Arabic authors. As often photographs of the authors are available, gender recognition was in many cases rather straightforward.

With these methods, we could assign gender to 94% of the authors in our dataset, and to more than 98% of the article shares. We were not able to assign gender in cases where (i) none of the articles of an author had a full first name; (ii) authors with Asian names where no web information could be found—as it is often impossible to detect gender using names that were translated to western characters.

### Personal characteristics

Bibliometric data do include only a few personal attributes in the publication’s metadata. For a sample of our set, we were able to collect data about academic position and for age, enabling to investigate the effect of these two covariates. We used for this university personnel registers of ten of the largest Swedish universities (Chalmers, Gothenburg, Karolinska, KTH, Linkoping, Lund, SLU, Stockholm, Uppsala and Umea) covering the 2004–2008 period. That provides age and position information about for about 6,000 researchers who published in the 2008–2011 period. Furthermore, from the bibliometric data, we are able to calculate other variables, e.g. the research field (each person has been assigned to the field where most of the publications are published over the period) and the number of last authorships reflecting the position in the research team or collaboration network.

Most variables are straightforward, but a few need more explanation: For academic position we distinguish the following: (i) all professor positions, like full professor, endowed professor, adjunct professor, acting professor, etc.; (ii) university docent, which is mainly a teaching position; (iii) researcher, including researching medical doctors in medical schools; (iv) postdocs, including research assistants, and (v) PhD students. This classification is not the same as the categories distinguished in Fig 1, as universities use different categories than Statistics Sweden. The number of last authorships were calculated by the ‘author order mechanism’ in the BMX program.

### Indicators

Only when a paper is highly cited, it can be considered as really contributing to scientific progress [26]. Until recently, quality of an oeuvre was often defined in terms of *size-independent* indicators: the *percentage* of highly cited papers within the oeuvre of an author. This implies that small and large oeuvres can be equally good. However, there is a move towards *size-dependent* indicators, as the *number* of highly cited papers of an author indicates the contribution to scientific progress and that number creates visibility in the scientific community. This choice of size dependent indicators influences the position of female researchers, as it is often claimed although that women publish less than men do, they tend to be cited equally well [9; 7] or even better [33]. This would imply that female researchers may have the same share of top papers in their oeuvre as men have, but a lower absolute number.

### Methods

1. Similarly to Sandström & Van den Besselaar [4], we investigate whether the *number* and *share* of CSS3 or CSS1 papers increases with productivity. We first calculate the average number of CSS3 papers, given the productivity level of authors. In order to do so, we classified all 45.000 Swedish researchers (all those assigned a gender) into productivity classes: productivity class 1 has one publication in the four years period under study, class 2 has two, class 3 has three to four, class 4 has five to eight, class 5 has nine to sixteen, class 6 has seventeen to 31 publications, and finally class 7 covers researchers with 32 or more publications. The bins have been sized to be roughly even on a logarithmic scale. For each of these classes, we calculate the average number of papers in the CSS3 class. This is done for the whole set, and for the eight research fields separately, based on the mentioned classification [34], and with the humanities field added by us. Publications are integer counted, and citations are field normalized. The *average number of papers in CSS 3* is not an integer, as on average only one out of some 30 papers belongs to CSS3.
2. We do a regression with the total number of (fractional counted) publications of an author as the independent variable, and the (also fractional counted) number of top cited publications of the same author (for each of the definitions of ‘top cited’ that were discussed above). Likewise, citations are field normalized. This is done for the total population of researchers and for the male and female subpopulations separately
3. We calculate the percentage of women in the various productivity classes by field, in order to get a more detailed picture of the productivity differences. This analysis shows indeed a difference which is explained in the next step.
4. We do regression analysis with the fractional counted publications as dependent variable and gender as independent variable. We then redo this using a series of covariates: we control for age, field, academic position, the number of last author positions. Comparing the regression coefficients of gender in the two regressions informs us about (i) the effect of gender on productivity, and (ii) what remains of the effect of gender when controlling for the other variables. As the data are over-dispersed, we use from the generalized linear models suite the *negative binomial with log link model*. The latter model works only with count data (integers), and therefore we multiplied the fractional score with 1000 and rounded it up to the nearest integer.

## Results

### Gender differences in the relation between highly-cited papers and productivity?

**Fig 3:**
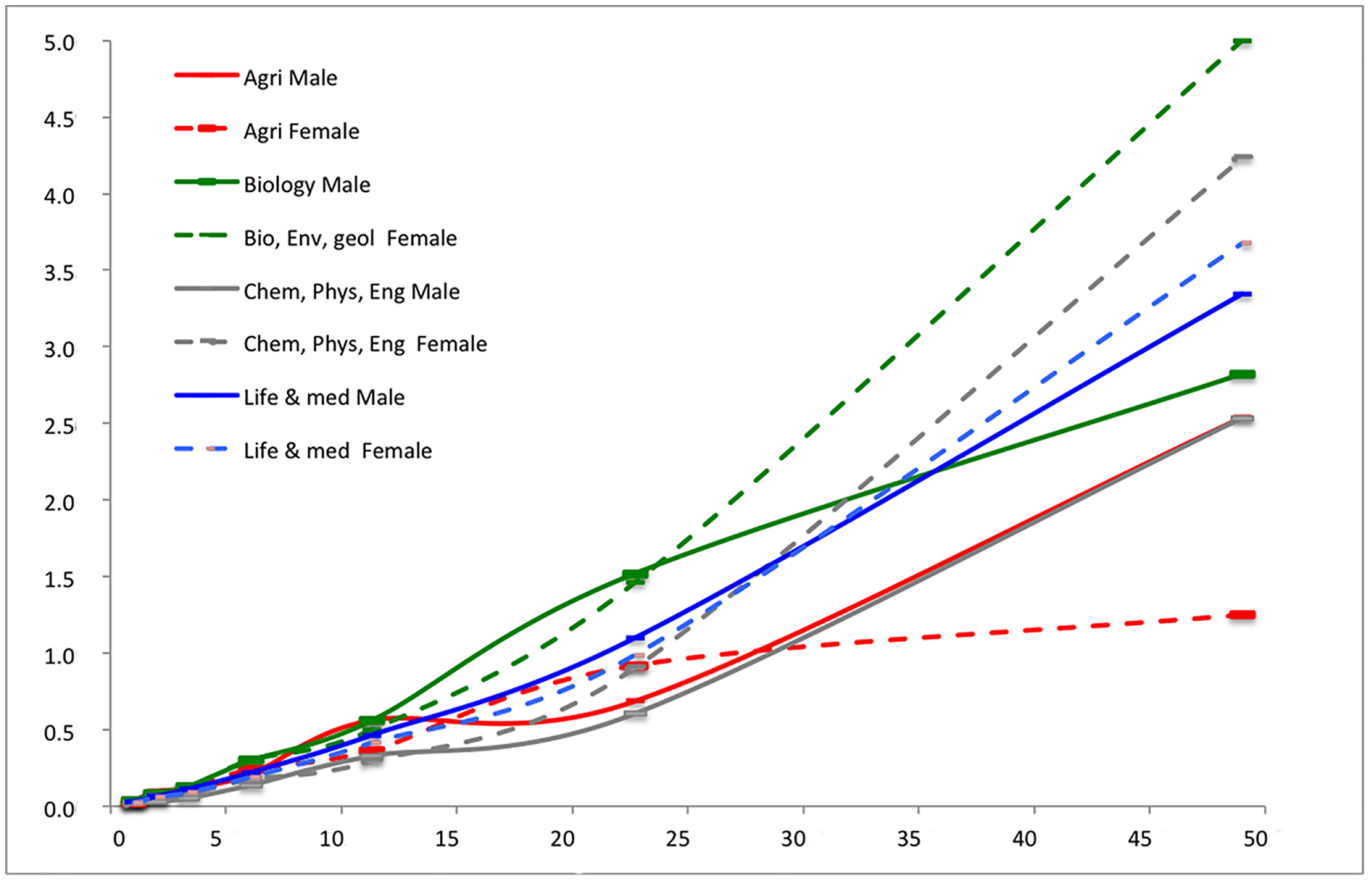
Number of CSS3 papers by productivity class and discipline. Male researchers = straight line, female researchers = dotted line; Lines connect the data points of the seven productivity classes.

**Fig 4:**
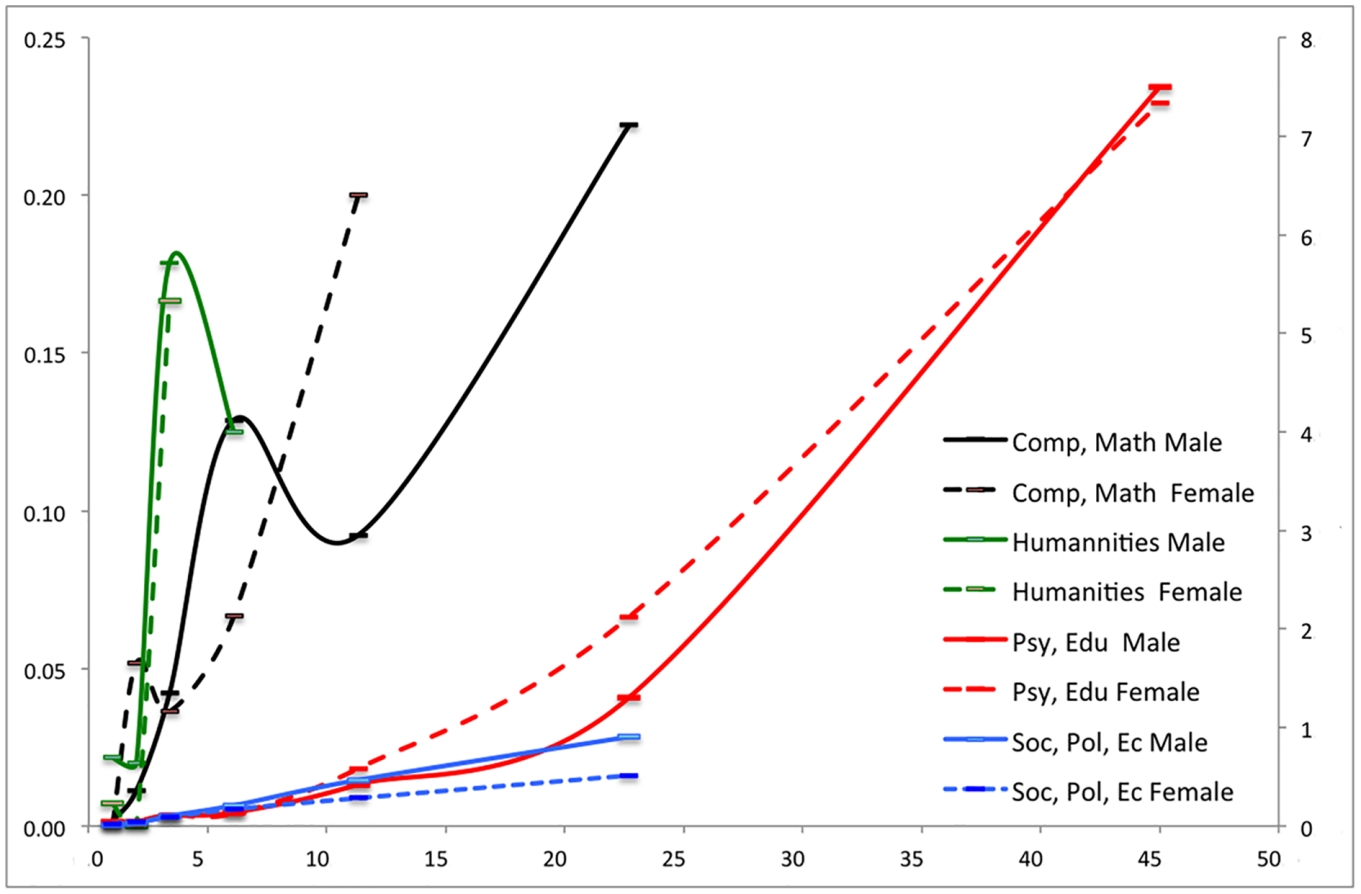
Number of CSS3 papers by productivity class and discipline. Male researchers = straight line, female researchers = dotted line; Lines connect the data points of the seven productivity classes.

We calculated for each of the seven productivity classes and each of the eight research domains the average number of top cited papers within the CSS3 class. Figs 3 and 4 show the relation for the eight fields for male and female researchers. In Fig 3, we present the data for the “Science & Engineering”–men and women, and in Fig 4 for the “Social Sciences”, the “Humanities” and for “Computer Science & Mathematics”—also male and female researcher. This way of presentation enables the comparison.

All fields but two show a positive relation between the share of CSS3 papers and productivity; exceptions are the fields *Computer science & mathematics* and *Humanities* (male researchers), where is more fluctuation within the overall positive trend. It should be noted that in these two fields the numbers are relatively low, as scholarly articles are not the dominant form of publishing. In some fields, the relation between productivity and the number of CSS3 papers is constant, as is shown by a straight line (e.g., *Social sciences*), but in other cases the number of CSS3 papers increases more than linear with output.

What about the gender differences? In *Biology*, *Life & Medical sciences* and in *Science and Engineering*, women in the higher productivity classes outperform the male researchers, as they have on average a higher number of CSS3 papers: the dotted curves (representing female researchers) for these fields are above the straight curves (representing male researchers). Also in *Psychology & Education* we see such trend, although in the highest productivity class the scores are equal. In *Agriculture and Food Sciences*, and in the *Social Sciences*, the pattern is opposite. As already said, in the *Humanities* and in *Computer Science & Mathematics* the pattern is somewhat fuzzy, but in the latter field there are no female researchers in the highest productivity class to compare with male counterparts.

Overall the figures show that both for men and women, the number of CSS3 papers increases absolutely and relatively with productivity, and quality and quantity of output highly correlate. As Figs 3 and 4 show, some gender differences were found. Comparing average impact of female and male researchers *within the productivity classes* we found that female researchers perform on average substantially better in the *sciences & engineering*, in *biology*, *environmental sciences & geology*, and in computer science & mathematics. In *agriculture*, and in the *social sciences* it is the opposite. In *life sciences & medical sciences*, in *psychology*, and in the *humanities*, no gender differences were found *within* the productivity classes.

Why these disciplinary differences exist needs further investigation, but it seems that the lower the percentages of women in a field, the better they perform compared to men within the same productivity class (Table 2). This could either between the entrance for women in those fields is more difficult (gendered selection: they need higher performance than men to be able to enter the field), or that women only if they are really good enter such fields (gendered self-selection). Only psychology diverges from this pattern.

**Table 2:** Gendered performance differences by gendered discipline demography.

Discipline	Male/female researchers in the field	Top cited papers/all papers
Psychology	0.78	equal
Social sciences	1.06	male > female
Agriculture	1.06	male > female
Life sciences & medicine	1.18	equal
Humanities	1.49	equal
Biology & geology	1.70	female > male
Sciences & engineering	3.05	female > male
Computer science / math	4.26	female > male

### The relation between productivity and the number of high-cited papers

**Fig 5:**
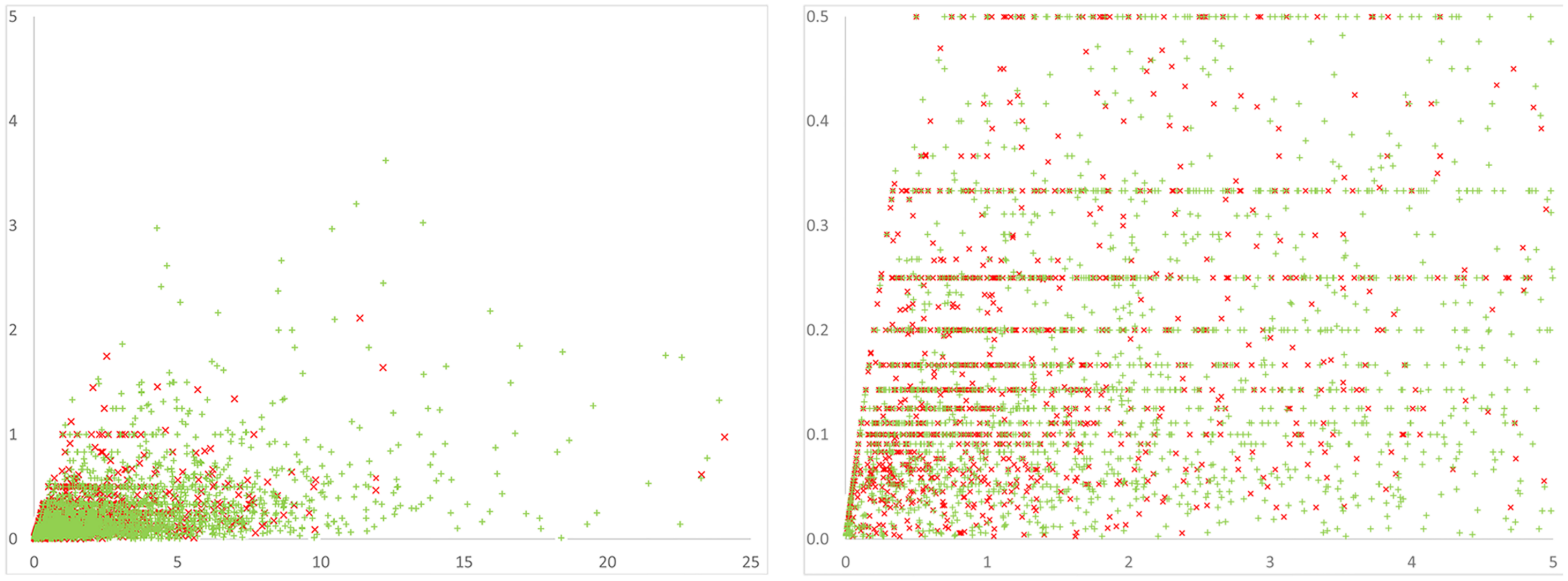
Number of CSS3 cited papers by total number of papers and gender. Left figure: all authors. Right figure: enlarged dense area with low productive authors. Red = female; green = male. Papers and CSS3 papers both fractionally counted. Based on 44,500 Swedish researchers with assigned gender, publications 2008–2011, and citations until end of 2015.

In the previous section we compared male and female productivity within the productivity classes—here we do an overall analysis of the relation between productivity and impact for men and women. In [4] we did so without including gender, and used a variety of definitions of ‘top cited publications’ (1% most cited publications, 10% most cited publications, CSS3, CSS1), integer counted, fractional counted and with field adjusted production. All analyzes point in the same direction: more papers lead to (proportionally) more top cited papers. In Fig 5 we show one example distinguishing between male and female researchers: the number of CSS3 papers by productivity (both fractional counted). The right figure shows the large amount less productive researchers, as this part of the plot is too dense to inspect in the left figure. The right figure suggest that the red and green dots are reasonably well mixed, with a tendency of more red dots above the diagonal. This indicates that female researchers perform *at a specific productivity level* at least equally well as male researchers do. However, the left figure shows the complete scatter plot, and the red dots (representing female researchers) are underrepresented at the higher productivity levels where one observes mainly green dots (male researchers). So overall, male researchers are more productive than female.

Calculating the correlations between the number of papers and the number of top cited papers shows that at the CSS1 level the relation between quality and quantity is fairly strong: 0.88 for full counted, and 0.80 for fractionally counted papers (Table 3). For men and women separately we find about similar correlations for women (0.78) and for men (0.81). At the CSS3 level, the patterns are the same but the correlation between the number of papers and top-cited papers is moderately strong. Only about 10% of the authors do have CSS3 papers, as productivity has a skewed distribution, for example the 6.3% most productive authors are decisive: they have 49% of the fractional counted CSS3 papers. The existence of low productive authors with high cited papers may be the effect of increasing teamwork in research: a PhD student or postdoc can be the author of a very good paper, as member of the team, but him/herself not very productive over the longer period. Also Larivière & Costas [5; 6] suggested that the relation between quality and quantity typically holds for the more experienced (and more senior) researchers.

**Table 3:** Correlation between the number of papers and the number of top papers.

	Full counted	Fractional counted		
	All	All	Female	Male
CSS3	0.58	0.43	0.40	0.43
CSS1	0.88	0.80	0.78	0.81

### Differences in gender distribution over the productivity classes

**Fig 6:**
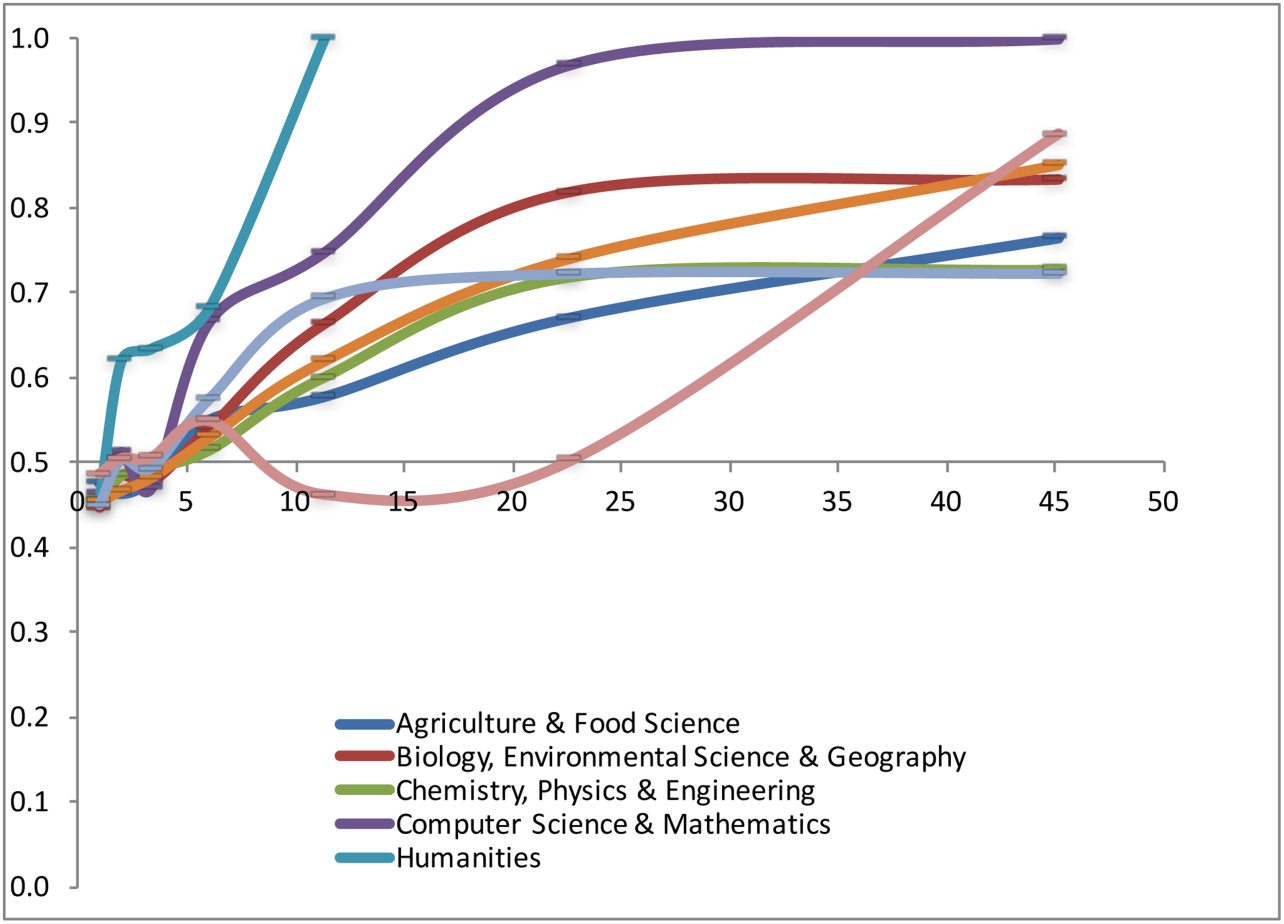
Gender by productivity category and field. (Vertical axis: share of men; horizontal axis: average number of papers for each of the productivity groups; lines connect the data points of the seven productivity classes.)

The relation between productivity and quality/impact is not gender specific, as the above analysis suggest. But we do still observe differences, as the overall productivity of female researchers in on average 67% of productivity of male researchers. This is a rather constant finding over various decades, and earlier studies found similar ratios (see references in the Introduction). In fact, it is substantially lower than the 1993 value found by Xie & Schauman [9], so there is no long-term decline that we discussed in the introduction of this paper. Fig 6 is disaggregated to eight research domains, and gives a more differentiated picture of the relation between the share of male researchers and the share of female researchers in each of the productivity categories. The Y-axis represents the share of men and women in a field and productivity category (M/M+F). If the share of male and female is equal, the value is 0.5. If the value is above 0.5 then more men are in that productivity category. And if the score is 1, only men are in that productivity category. The overall the pattern is clear: in the lower productivity categories, there are about an equal number of male and female researchers, but the higher the productivity category, the larger share of men, and the lower the share of women in that category.

### Explaining gender differences in productivity

How can this be explained, that women are strongly underrepresented in the high productivity groups? In the introduction, we introduced several possible factors that may explain the productivity differences. ANOVA shows that gender correlates with the other independent variables, as men are on average 3.5 years older (p = .000), 75% more last author (p = .000), and have on average a higher position, but on average have an equal number of co-authors.

In order to test the effect of these factors, we conducted a regression analysis, in which we investigate the effect of gender on productivity, whereas controlling for age, academic position, position in research group (with author position as proxy), research field, and age.

Table 4 shows that female researchers have on average significantly less fractional counted publications, with a regression coefficient is -0.431. But, when we control for several other variables (see Table 5), the regression coefficient for gender goes down to about one third of the original value (-0.148). When controlling for research field, the other independent variables university position, last author position, and the number of co-authors, contribute all to gender differences in productivity, as was expected from the literature. These variables mediate the effect of gender on productivity: gender influences the variables like academic rank and position in research collaboration and in teams, and these in turn have an effect on productivity, which may reciprocally influence academic rank and the role female researchers have in their team. One should note that age has disappeared from the model, as age had no effect whatsoever. This is because university position (status) correlates very strongly with age.

**Table 4:** Model 1—Productivity by gender.

	B	Std. Error	95% Wald Confidence Interval		Wald Chi-Square	df	Sig.
			Lower	Upper			
(Intercept)	7.549	.0167	7.516	7.582	204067.692	1	.000
Gender: female vs male	-.431	.0283	-.487	-.376	232.365	1	.000

Dependent Variable: Fractionally counted publications

Model: (Intercept), Gender; Goodness of fit: 1.131

**Table 5:** Model 2—Productivity by gender, academic position, field, last author position, and number of coauthors.

	B	Std. Error	95% Wald Confidence Interval		Wald Chi-Square	df	Sig.
			Lower	Upper			
(Intercept)	6.826	.0472	6.733	6.918	20952.063	1	.000
Gender: female vs male	-.148	.0295	-.206	-.090	25.167	1	.000
Position: prof vs PhD student	.277	.0439	.191	.363	39.813	1	.000
Position: docent vs PhD student	.179	.0429	.095	.263	17.429	1	.000
Position: researcher vs PhD stud.	.214	.0400	.135	.292	28.517	1	.000
Position: Postdoc vs PhD student	.329	.0518	.228	.431	40.436	1	.000
Research field: AGR vs SOC	-.183	.0717	-.324	-.043	6.524	1	.011
Research field: BEG vs SOC	.002	.0573	-.110	.114	.001	1	.969
Research field: COM vs SOC	-.106	.0681	-.239	.027	2.423	1	.120
Research field: CPE vs SOC	-.022	.0503	-.121	.076	.195	1	.659
Research field: LIFE vs SOC	-.193	.0442	-.280	-.107	19.124	1	.000
Research field: PSY vs SOC	-.072	.0665	-.202	.058	1.172	1	.279
Leading (last) author position	.162	.0049	.152	.171	1085.278	1	.000
Number coauthors	-.016	.0015	-.019	-.013	101.476	1	.000

Dependent Variable: Fractionally counted publications; Model fit: 0.573. AGR = agriculture; SOC = social sciences; BEG = biology, ecology, geology; COM = computer science and mathematics; CPE = chemistry, physics, engineering; LIFE = medical and life sciences; PSY = psychology

## Conclusions

We used a large dataset with variables that have hardly been taken into consideration in studies on gender productivity differences. That resulted in the following findings:

The first question we aim to answer is whether the positive relation between productivity and impact differs between male and female researchers. We showed that this is not the case, and the relation between productivity and the number of high impact papers is about the same for men and women within the distinguished productivity classes. On average, female researchers have a at least similar impact as equally productive male researchers. In fact, we found cases where the ratio between top cited papers and productivity is considerable higher for women than for men. More specifically, the disciplinary demography seems to produce this effect: the lower the share of women in a discipline, the higher their impact compared to male researchers within the same productivity class. This may refer to gendered selection and/or to gendered self-selection.

Secondly, we found that the higher productivity classes are numerically dominated by male researchers. This leads to a lower overall productivity for female researchers, which is also in our sample about 70% of male productivity. This ratio seems to be stable over time. We should however be careful with averages in Lotka distributed data, although nonparametric tests (Mann-Whitney) show that women are outperformed by male researchers is we do not take other factors into consideration.

Thirdly, we investigated whether other variables influence productivity, and therefore explain part of the gendered productivity differences. We indeed found that a variety of factors have an effect on performance, and controlling for those reduced the effect of gender on performance considerable. So, a good part of the productivity differences are due to the fact that men are older and in higher positions, and that those in higher positions are more productive. Female researchers also occupy less last author positions than men do, and this factor also has a negative effect on female productivity. That women more often are in the middle author positions than men, reflects that women have on average lower positions, and that they are less often (conceived as) leader of a team or a collaboration network. This finding reflects that male researchers show a faster career than their female counterparts.

Summarizing, gender influences the academic rank, and the role of researchers in research teams and networks: female researchers tend to have lower academic positions, and tend to are less often in a leading role. This then has a negative effect on the performance of female researchers, which in turn reinforces the lower status and position. If this is correct, female researchers more often than male researchers are caught in this *vicious cycle*, which may explain the persistence of the *glass ceiling*. It is not difficult to understand why gender differences in getting positions in top research environments, and gender differences in getting (prestigious) research grants may make this self-reinforcing dynamic even stronger.

**Fig 7:**
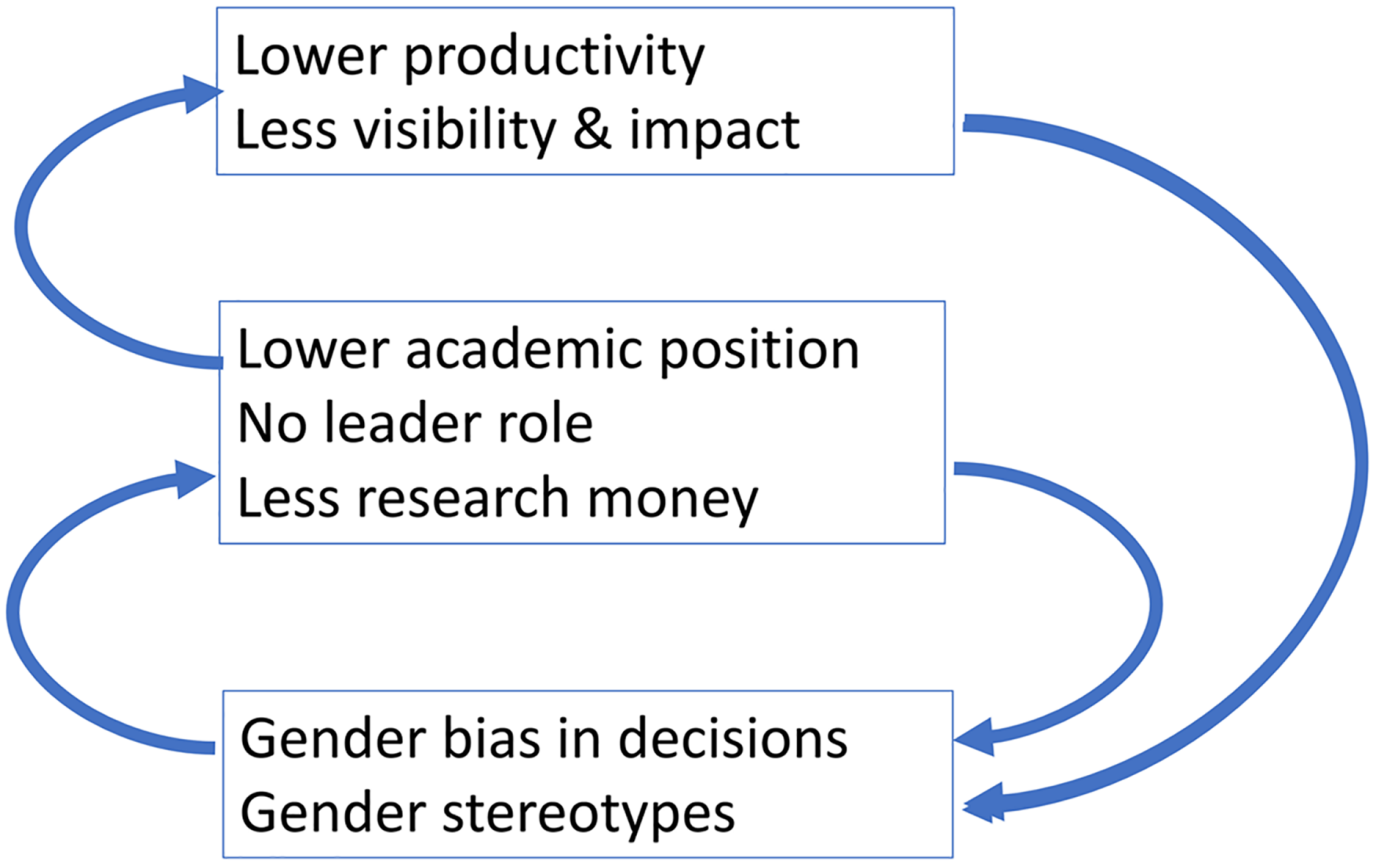
Gender bias as coupled vicious circles.

Overall, this suggests that several factors together may disadvantage female researchers, as these have less opportunities to develop into a high productive researcher. Accordingly, it cannot be expected that the differences between male and female researchers will simply diminish over time as earlier observed trends may suggest. Therefore, gender equality policies remain important to reverse the coupled vicious circles that produce the glass ceiling (Fig 7).

A limitation of this study is that some of the explaining factors were not included in the analysis, due to lack of data: (i) gender differences in the quality and research intensity of the research environment; (ii) gender differences in getting grant applications funded; (iii) other aspects of career, like gender differences in having a permanent position. Another limitation is that the data are only about the Swedish research system. However, we have no reasons to expect that Sweden is in this respect different from other advanced science countries. Both limitations at the same time point at the directions for further research.
